# Improving Stress and Positive Mental Health at Work via an App-Based Intervention: A Large-Scale Multi-Center Randomized Control Trial

**DOI:** 10.3389/fpsyg.2019.02745

**Published:** 2019-12-06

**Authors:** Silvana Weber, Christopher Lorenz, Nicola Hemmings

**Affiliations:** ^1^Human-Computer-Media Institute, Julius-Maximilians-University, Würzburg, Germany; ^2^Soma Analytics, London, United Kingdom

**Keywords:** stress, work, RCT, mental health, digital health, mobile health intervention, smartphone app

## Abstract

Mobile health interventions (i.e., “apps”) are used to address mental health and are an increasingly popular method available to both individuals and organizations to manage workplace stress. However, at present, there is a lack of research on the effectiveness of mobile health interventions in counteracting or improving stress-related health problems, particularly in naturalistic, non-clinical settings. This project aimed at validating a mobile health intervention (which is theoretically grounded in the Job Demands-Resources Model) in preventing and managing stress at work. Within the mobile health intervention, employees make an evidence-based, personalized, psycho-educational journey to build further resources, and thus, reduce stress. A large-scale longitudinal randomized control trial, conducted with six European companies over 6 weeks using four measurement points, examined indicators of mental health via measures of stress, wellbeing, resilience, and sleep. The data were analyzed by means of hierarchical multilevel models for repeated measures, including both self-report measures and user behavior metrics from the app. The results (*n* = 532) suggest that using the mobile health intervention (vs. waitlist control group) significantly improved stress and wellbeing over time. Higher engagement in the intervention increased the beneficial effects. Additionally, use of the sleep tracking function led to an improvement in sleeping troubles. The intervention had no effects on measures of physical health or social community at work. Theoretical and practical implications of these findings are discussed, focusing on benefits and challenges of using technological solutions for organizations to support individuals’ mental health in the workplace.

## Introduction

In recent decades, work-related stress has become increasingly prominent due to changes in working conditions (e.g., Kompier, 2002; Landsbergis, 2003; Broughton, 2010). Technology represents a potential solution to the issue of workplace stress, as smartphone technologies have become a common part of daily living (Bolier and Abello, 2014). Accessible, scalable, and providing return on investment (Ebert et al., 2014, 2016), mobile health interventions (i.e., mental health apps) may drive positive behavior change and are becoming increasingly available for individuals and organizations to manage mental health, both in terms of treatment and prevention programs. However, researchers, clinicians, and practitioners alike have identified both a paucity of empirical evidence for the effectiveness, and a lack of congruence with scientific theories and guidelines of mobile health interventions (e.g., Webb et al., 2010; Donker et al., 2013; Bolier and Abello, 2014; Nicholas et al., 2015; Bakker et al., 2016; Howells et al., 2016; Mistretta et al., 2018).

The current project addresses this research gap by investigating the effectiveness of a science-backed mobile health intervention to manage stress and positive mental health^1^ at work. As part of a European Union’s Horizon 2020 research and innovation project, this paper describes findings of a large-scale, multi-center randomized control trial (RCT) to assess a smartphone-delivered health intervention to counteract workplace stress and increase wellbeing at the individual level within a naturalistic, occupational context.

### Stress and Resilience in the Workplace

Workplace stress remains a common yet undertreated condition (Ebert et al., 2016). If experienced over a prolonged period of time, stress can result in a variety of mental and physical health issues (Joyce et al., 2016), affecting both the individual and the organization. Concerning the former, excessive chronic workloads without time to rest can cause physical exhaustion, stress-related illnesses (Chandola et al., 2006), and psychological disorders such as depression (Hammen, 2005). As to organizations, this results in high costs. From 2008 to 2017, sickness absence days due to mental ill-health^2^ increased by 67.5% in Germany (Meyer et al., 2018). In the United Kingdom, in 2018, the Health and Safety Executive (2017) reported that stress, depression, and anxiety accounted for 15.4 million working days lost due to ill-health (57% of all days lost). Similar figures have been reported for other European countries (European Agency for Safety and Health at Work, 2014).

To counterbalance the risks that come with high levels of work stress, the concept of employee *resilience* has become increasingly important. Definitions of resilience vary, regarding it either as a rather stable trait that helps individuals to cope with difficulties and to attain good adjustment and development (e.g., Wagnild and Young, 1993; Schumacher et al., 2005; Ong et al., 2006), or as a state, suggesting a dynamic process, in which individuals actively adapt to and recover from major difficulties (e.g., Luthar and Cicchetti, 2000; Connor and Davidson, 2003; Fergus and Zimmerman, 2005; Windle, 2010). Taking the perspective that resources to counteract stress are adaptable, the *Job Demands-Resources Model* (JDR; Bakker and Demerouti, 2007; Schaufeli and Taris, 2014) describes stress as a response to an imbalance between the demands that work places on the individual and the resources a person has available to deal with those demands. *Job demands* describe “physical, social, or organizational aspects of the job that require sustained physical or mental effort and are therefore associated with certain physiological and psychological costs” (Demerouti et al., 2001, p. 501), for example, work overload, interpersonal conflicts, or job insecurity (Schaufeli and Taris, 2014). *Job resources* are defined as “those physical, psychological, social, or organizational aspects of the job that may do any of the following: (a) be functional in achieving work goals, (b) reduce job demands at the associated physiological and psychological costs; and (c) stimulate personal growth and development” (Demerouti et al., 2001, p. 501), resulting in higher employee resilience. If demands exceed resources, individuals experience a mental and physical health impairment process, leading to decreased energy and exhaustion. If the contrary is the case, a motivational process takes place, with an increase in work engagement and positive outcomes such as higher wellbeing and organizational commitment (e.g., Bakker et al., 2003; Schaufeli and Bakker, 2004; Xanthopoulou et al., 2007; Hakanen et al., 2008).

### Digital Mental Health Interventions: Categorization, Scientific Foundation, and Validation

In order to tackle problems associated with stress in people’s everyday lives, the *mHealth*^3^ sector, and with it, the availability of apps claiming to reduce stress, increase wellbeing, or improve mental health issues such as depression, is constantly growing (e.g., Muaremi et al., 2013; Research 2 Guidance, 2017; Statista, 2018). Such mobile health interventions can be classified into four categories (Muaremi et al., 2013): diaries (collection of subjective and aggregated data), guides (strategies to cope with the problems), relaxations (training of relaxation skills), and sensor measurements (sensor-based tracking of problem-associated behavior). Many stress and wellbeing apps include features from more than one of these categories. Mobile health interventions can provide personalized feedback and deliver summary statistics or progress scores, either via the app or transmitted through the app by a therapist or coach (e.g., Gaggioli and Riva, 2013; Ly et al., 2014; Heber et al., 2016; Firth et al., 2017). Research demonstrates that the use of scientific methods and a theoretical foundation positively influence the outcomes of technologically delivered health interventions and drive behavior change (e.g., Webb et al., 2010; Donker et al., 2013; Bolier and Abello, 2014).

Recent research has shown that there is a large discrepancy between using science to advertise mental health apps versus evaluating their effectiveness (Larsen et al., 2019). Few of the many mobile health interventions currently on the market are scientifically validated; as such, the quality of mental health apps differs widely regarding the level of scientific evidence (e.g., Bolier and Abello, 2014; Nicholas et al., 2015). Meta-analyses show that online interventions (e.g., internet-delivered cognitive behavioral therapy) can be equally as effective as traditional face-to-face interventions in treating psychological disorders (e.g., Barak et al., 2008; Carlbring et al., 2018). A comparable level of research on mental health apps is lacking. In the clinical domain, some apps have been successfully applied concerning the diagnosis and treatment of stress-related mental illnesses such as depression and anxiety (e.g., Watts et al., 2013; Birney et al., 2016; Ranjbartabar et al., 2016; Firth et al., 2017). Yet, there remains a need for more rigorous research on the effectiveness of mobile health interventions as prevention tools for mental health, particularly in the work context.

### Preventative Mental Health Solutions in the Work Context

Whilst research on digital health interventions in the clinical domain is rapidly growing (Firth et al., 2017), research on preventative mental health solutions in the workplace is often overlooked. Although workplaces would constitute an ideal location for preventative programs, most organizations implement reactive measures targeting the symptoms of workplace stress (Deloitte, 2017). Previous workplace mobile health interventions are built on programs traditionally run in a clinical context, such as cognitive behavioral therapy (CBT) or mindfulness based cognitive therapy. They often center around a ‘virtual coach’ or councilor guiding the user through content. Some pioneering studies found positive effects for smartphone interventions on workplace stress, for example, using Acceptance and Commitment Therapy (ACT; Ly et al., 2014) or CBT (Joyce et al., 2016). Initial results look promising; however, characteristics of the target population have to be considered when transferring tools to support mental health from a clinical domain to the workplace population (Joyce et al., 2016).

Mobile health interventions provide an opportunity for the provision of a proactive, preventative approach for employee mental health (e.g., Tan et al., 2014; Aryana et al., 2018). They are easily accessible to employees, enabling access to a service earlier than traditional methods, and therefore, preventing the onset of more severe mental health problems (Ebert et al., 2016). A mobile preventative intervention approach may lend itself to higher levels of personalization than traditional workplace wellbeing support. Through a digital pathway, employees are able to take control of their individual journey by completing the intervention at their own pace, working on content that applies to them and their personal situation, and choosing a time that suites them while staying in anonymity (cf. Gaggioli and Riva, 2013; Ebert et al., 2016; Joyce et al., 2016). In sum, this creates an environment of high opportunity and low demand in which the desired behavior change can occur, leading to improved psychological outcomes (cf. Howarth et al., 2018). This may not only benefit the individual, but also the organization as a whole.

Programs specifically focusing on work-based problems often link to daily work routines (e.g., commuting or eating), and consist of targeted, short, simple, and easy to implement tasks, providing an opportunity to easily transfer learnings into daily work life (e.g., Gaggioli and Riva, 2013; Ebert et al., 2016; Joyce et al., 2016). Mobile health interventions benefit further when embedded into the work environment, for example, when downloaded onto an employee’s personal technical equipment such as their work phone (Howarth et al., 2018; see also Martinez and Williams, 2010). Smartphones are commonly used among people of different age groups, socio-economic status, and cultural backgrounds; thus, app-based health interventions can be made accessible to a broad range of people in different workplaces. Often, users carry their smartphones almost everywhere and anytime (He and Li, 2013), providing a range of measurement and intervention opportunities, such as real-time symptom and activity monitoring, tracking of treatment progress, provision of personalized feedback and motivational support, portability and flexibility of use, and the potential to improve adherence to treatment (Donker et al., 2013). Furthermore, users may be empowered by the feeling of privacy and confidentiality of their engagement, if they only interact with an app on their personal phone, without connections to social networking sites (Bakker et al., 2016); still, data security policies need to be ensured.

### The Current Research

Taken together, theory and research suggest that mobile health interventions may help to prevent mental health problems due to work-related stress, provided the intervention is grounded in theory, uses evidence-based techniques, and implements various behavior change strategies (e.g., based on self-monitoring or dual process theory). There is promise in regards to future science-backed mobile health interventions, as research in this domain is on the rise (e.g., Deady et al., 2018). Yet, there is still a lack of experimental research to validate app-based interventions to reduce stress at work.

The aim of this research was to examine whether a science-based health and wellbeing application, named “Kelaa Mental Resilience” and provided by Soma Analytics (London, United Kingdom), drives statistically and functionally significant improvements in validated measures of stress and wellbeing. The app is a digital tool that focuses on the prevention of mental ill-health, rather than a treatment thereof. It is theoretically grounded in the JDR (e.g., Demerouti et al., 2001; Bakker and Demerouti, 2007). The mobile health interventions has been developed specifically for the workplace and offers a combination of diaries, sensor measurements, and guides (see Muaremi et al., 2013). Details are provided in the paragraph *Kelaa Mental Resilience App* in the section “Materials and Methods.” A previous version of the app was already shown to be effective for healthcare workers in a clinical work context based on a small-scale RCT (Mistretta et al., 2018).

Within the European Union’s Horizon 2020 Research and Innovation Program, we conducted a large-scale longitudinal RCT to assess the intervention impact by comparing health-related outcomes between the app group and a waitlist control group. To increase external validity and generalizability of the findings, the trial was implemented at various organizations, offering a broad range of different jobs and work environments. Indicators of stress (cognitive and general), wellbeing, resilience, and sleeping troubles served as dependent variables (DVs). Further, we analyzed whether the intervention impact was influenced by other factors, such as intensity of engagement with the app over time or trial site.

Based on the theory and research outlined above, we expected the following:

*Hypothesis 1:* Compared to the waitlist control, after using the app for 4 weeks, participants in the app group will report (a) lower levels of stress (cognitive and general), (b) higher levels of wellbeing, (c) higher levels of resilience, and (d) fewer sleeping troubles.

*Hypothesis 2:* The observed effects will be more intense the more the user interacts with the app throughout the duration of the study.

In addition to the *a priori* hypotheses, we explored two open research questions: First, does the app also affect other outcomes which are more remote to the content of the app, such as social community at work (i.e., a measure of the organizational climate) or physical health? And second, will the positive effects of the app persist after people cease using it? To examine potential long-term effects, we included a 2-week follow-up measurement occasion (i.e., a time point after the main part of the study when participants had stopped using the app).

## Materials and Methods

### Kelaa Mental Resilience App

The app, developed as a digital prevention tool, seeks to translate insights from scientific research on psychology, sleep medicine, and neuroscience into an action-based program. It draws on the tenets of clinical, health, positive, cognitive, biological, and social psychology to foster recovery and growth. “Kelaa” aims to reduce stress and increase wellbeing of the user, specifically in the workplace. Users learn new behaviors and best practices through different means, for example, based on CBT and mindfulness based cognitive therapy. The app is designed to implement lifestyle changes through (1) measuring behavior, cognitions, and emotions (tracking module) and (2) providing psycho-educational content (intervention module).

Within the tracking module, users can track their stress, wellbeing, and resilience via short in-app questionnaires using validated scientific measures. The app also uses inbuilt sensors in smartphones (e.g., the accelerometer) to provide the opportunity to measure and track their sleep quality and quantity. Personalized feedback on questionnaire scores (e.g., what are my scores? What does this mean for me? What should I do about this?), as well as detailed feedback on sleep data (e.g., how do I interpret my sleep charts?), are given within the app.

In the intervention module, users access structured science-based content on factors contributing to reduced stress and improved wellbeing. “Kelaa” provides the user with evidence-based interventions grounded in current research, for instance, from sleep science, positive and social psychology, self-monitoring, CBT, and mindfulness. According to the *Hedonic Adaptation Prevention Model*, task variety is a prominent factor influencing the effectiveness of happiness interventions, especially in the long-term. Engaging in a larger variety of exercises results in greater benefits from the intervention, causing an additional increase in wellbeing both in the short and long term (Sheldon et al., 2013). Building upon these findings, “Kelaa” offers users the opportunity to choose from a variety of topics of interventions, based on their individual results from the tracking module and personal interest. This individual choice is also intended to stimulate higher intrinsic motivation. Then, the user journeys through self-selected goals on different content. Each goal includes six to seven “daily sessions” (each about 2–4 min to read), which are gradually unlocked. The goals aim to increase personal resources by providing information, exercises, and reflection. During each daily session, relevant research and expected benefits are outlined, before users are instructed, for example, in specific stress management and resilience techniques, while encouraging positive behavior transformation. The intervention module supports users to reach a variety of nominated goals. A detailed summary of all goals and sessions is provided in Table 1.

**TABLE 1:** Summary of topics and content that was available through the app as part of the intervention.

**Topic (“weekly goal”)**	**Content (“daily sessions”)**	**Percentage of all topics chosen**
Happiness	The pleasant, good and meaningful life; three good things; gratitude; using strengths in new ways	20.8%
Sleep	Pre-sleep routine; sleep environment; regularity; circadian rhythm; daylight exposure; artificial light	17.6%
Personal productivity	Prioritization; time management (e.g., “eat your frog”); Eisenhower matrix; Pomodoro technique; email batching; Pareto rule	15.0%
Energy and focus	Psycho-education on mindfulness; mindful eating; negativity bias; multi-tasking; free will as a resource; “not-to-do list”	12.5%
Rumination	Psycho-education on rumination; thinking traps; techniques for cognitive re-evaluation; social comparison; mindfulness	10.2%
Stress recovery	Psycho-education on demands and resources; active management of recovery; social connections; negative thoughts; re-evaluation using ABCDE model	9.6%
Positive relationships	Pro-social spending; acts of kindness; gratitude letter; forgiveness; social environment	8.2%
Work relationships	Lifecycle of a team; appreciation; extra-curricular activities; non-judgmental communication; kindness	5.9%
Shift work	Regulating the circadian rhythm: light, stimulants, nutrition, schedule, routine	0.2%

### Participants

An *a priori* sample size calculation (*G^∗^Power*; Faul et al., 2007), with a conservatively estimated main treatment effect of at least 5% for all parameters (small effect: Cohen’s *d* = 0.28, Cohen’s *f* = 0.14), assuming a significance level (alpha) of 5%, and a statistical power (1-beta) of 80%, resulted in a required sample size of *N* = 561 participants. Participants were recruited from six different European businesses in Germany, England, and Northern Ireland from the private and public sector. The complete data set consisted of *N* = 678 participants. At T1, *n* = 621 people completed the questionnaire. The number of participants dropped to *n* = 483 at T2, *n* = 396 at T3, and *n* = 363 at T4. A total of *n* = 301 (44.4%) people completed all questionnaires at all times, while *n* = 105 people completed three (15.5%), *n* = 99 two (14.6%), and *n* = 146 (21.5%) only one out of four measurement occasions (see section “Procedure” for details).

As indicated by user metrics of the app, out of the *n* = 347 participants who were assigned to the app group, *n* = 137 people did not use the app at all, and thus, were excluded from the sample. Further, people in the waitlist control group (*n* = 331), who downloaded the app before the end of the trial (*n* = 9), were also excluded. All participants who adhered to their assigned group (i.e., app group with at least one sign-in, waitlist control group with no app use) were included into the statistical analyses.

The final sample consisted of *n* = 532 participants with *n* = 210 in the app group and *n* = 322 in the waitlist control group. Participants were unevenly distributed across trial sites (*n*_1_ = 40, *n*_2_ = 78, *n*_3_ = 179, *n*_4_ = 61, *n*_5_ = 11, *n*_6_ = 163). Participants’ age (*M* = 40.62, *SD* = 11.19) ranged between 17 and 72 years (based on *n* = 485 participants who shared their age). The gender distribution of the final sample was skewed with *n* = 119 (24.4%) male and *n* = 369 (75.6%) female participants (*n* = 44 preferred not to share their gender or selected “other”). The educational background (based on *n* = 489 participants who shared this information) in the sample was rather high (Primary/Middle school: 0.4%; Secondary school: 5.3%; High school/College: 17.6%; Bachelor/Undergraduate Degree: 34.2%; Master/Graduate Degree: 33.7%; PhD/Doctorate: 3.5%; Other: 5.3%).

### Procedure

This intervention study was conducted as a randomized control trial (RCT), following a longitudinal experimental design. Participation was voluntary. Data protection policies (i.e., GDPR) were strictly followed. Ethics approval was provided through the European Commission Horizon 2020 Ethics Appraisal Procedure (European Commission, 2018). Participants were blind to hypotheses and goals of the study, while HR managers were blind to each participant’s group assignment. HR managers had no insight into questionnaire results, app use intensity, or other personal information.

The trial was conducted from January until September 2018. Launch days varied between the six companies. After signing up and giving their informed consent, participants were randomly assigned to one out of two experimental conditions: app group vs. waitlist control. The trial was conducted over a period of 6 weeks. During the recruitment phase, employees were informed via email and information on a web portal that on launch day, they would receive an email containing a link to the first of the four assessments. Data was collected online using the survey software *Qualtrics*. Participants in the app group were asked to complete the first questionnaire prior to downloading and engaging with the app. Measurements (see section “Measures”) of all participants (i.e., app group and waitlist control group) were taken at baseline (T1, week 0), mid-intervention (T2, week 2), end-intervention (T3, week 4), and two-week follow-up (T4, week 6). Invitations to the follow-up questionnaires as well as reminders were sent via email. The time frame to complete each questionnaire was restricted to seven days. After finishing T4, all participants were thanked and fully debriefed. They had the opportunity to provide feedback on their personal experiences with using the app and to suggest improvements.

The duration of the intervention was 4 weeks. Thus, participants in the app group could complete a maximum of 28 sessions and track a maximum of 28 nights. As people prefer to receive self-help support materials on a familiar medium (see Martinez and Williams, 2010), participants were offered the choice to use the app on their personal or their work phones at their individual preferences. It was completely left to the user to what extent s/he wanted to engage with the app. Push-notifications were sent out as reminders, yet users had the option to turn them off. For the app group, active access to the interventional module within the app was withdrawn during the final 2 weeks, in order to get an indicator of whether and which gains from 4 weeks’ use persisted (T4). Participants in the waitlist control group received no intervention and no tracking opportunity for the duration of the trial (6 weeks), yet they had unrestricted access to treatment as usual within their companies. Upon completion of the trial, participants in the waitlist control group received access to the “Kelaa” app.

### Measures

Participants completed a series of questionnaires at all four measurement occasions (T1, T2, T3, and T4). At all times, reliability of the scales was good or excellent, as indicated by Cronbach’s α (see Table 2 for descriptive statistics). Scales were presented in the order as below. For each measurement occasion and scale, participants were instructed to answer the questions based on their experiences during the past 2 weeks. For all measures and all times, the mean response scale values were calculated.

**TABLE 2:** Descriptive statistics (M, SD, *n*) of all variables at times T1, T2, T3, and T4 based on the final sample.

		**Reliability**	**App group**		**Waitlist control**	
		**α**	***n***	***M (SD)***	***n***	***M (SD)***
Number of sessions completed			210	11.06 (7.34) Range: 1–28	322	0
Number of nights tracked			210	3.61 (6.11) Range: 0–25	322	0

General stress (4 items, scale range: 1–5)	T1	0.84	199	3.00 (0.76)	299	3.01 (0.73)
	T2	0.89	154	2.63 (0.77)	274	2.89 (0.77)
	T3	0.89	117	2.53 (0.82)	241	2.79 (0.81)
	T4	0.90	111	2.46 (0.80)	225	2.57 (0.81)

Cognitive stress (4 items, scale range: 1–5)	T1	0.83	199	2.59 (0.85)	297	2.63 (0.78)
	T2	0.87	154	2.36 (0.82)	274	2.50 (0.80)
	T3	0.88	117	2.25 (0.85)	241	2.48 (0.80)
	T4	0.90	111	2.17 (0.85)	227	2.34 (0.81)

Wellbeing (7 items, scale range: 1–5)	T1	0.86	199	3.26 (0.65)	295	3.23 (0.60)
	T2	0.88	152	3.39 (0.70)	273	3.30 (0.59)
	T3	0.89	117	3.47 (0.71)	241	3.33 (0.62)
	T4	0.93	111	3.45 (0.78)	227	3.44 (0.71)

Resilience (13 items, scale range: 1–7)	T1	0.89	199	4.95 (0.89)	292	4.98 (0.96)
	T2	0.90	150	5.08 (0.95)	270	5.05 (0.90)
	T3	0.93	115	5.14 (1.05)	238	5.10 (0.92)
	T4	0.93	111	5.26 (1.07)	227	5.26 (0.95)

Sleeping troubles (4 items, scale range: 1–5)	T1	0.87	199	2.71 (0.99)	292	2.83 (1.02)
	T2	0.88	150	2.41 (0.95)	269	2.76 (1.03)
	T3	0.90	115	2.37 (0.91)	237	2.60 (1.05)
	T4	0.90	111	2.22 (0.87)	227	2.54 (1.01)

Social community at work (3 items, scale range: 1–5)	T1	0.83	199	3.82 (0.76)	292	3.87 (0.75)
	T2	0.84	150	3.86 (0.85)	270	3.84 (0.72)
	T3	0.86	115	3.91 (0.77)	237	3.84 (0.73)
	T4	0.87	111	3.95 (0.78)	220	3.85 (0.76)

Physical health impairment (4 items, scale range: 1–5)	T1	0.93	199	1.94 (1.03)	292	2.07 (1.03)
	T2	0.94	150	1.97 (1.03)	269	1.94 (0.97)
	T3	0.93	115	1.97 (1.04)	237	2.01 (0.97)
	T4	0.95	110	1.83 (0.94)	227	1.93 (0.98)

#### Stress

Self-reported levels of stress were assessed with the two subscales *General Stress* (four items, e.g., “How often have you been stressed?”) and *Cognitive Stress* (four items, e.g., “How often have you had problems concentrating?”) from the *Copenhagen Psychosocial Questionnaire – Revised Version* (COPSOQ II; Pejtersen et al., 2010). The items were answered on a five-point scale (1 = *not at all*; 2 = *a small part of the time*; 3 = *part of the time*; 4 = *a large part of the time*; 5 = *all the time*). Higher values indicate more stress.

#### Wellbeing

Subjective wellbeing was measured with the *Warwick-Edinburgh Mental Wellbeing Scale* (Tennant et al., 2007). Seven items (e.g., “I’ve been feeling relaxed.”) were answered on a five-point scale (1 = *none of the time*; 2 = *rarely*; 3 = *some of the time*; 4 = *often*; 5 = *all of the time*). Higher values indicate more wellbeing.

#### Resilience

We assessed resilience with the *13-item Resilience Scale* (RS-13; Leppert et al., 2008), a short form of the Resilience Scale (RS-25; Wagnild and Young, 1993). Items (e.g., “I usually take things in stride.”) were answered on a seven-point scale (1 = *strongly disagree* to 7 = *strongly agree*). Higher values indicate more resilience.

#### Social Community at Work

Participants indicated their sense of cooperation and social community at work with the subscale *Social Community at Work* from the COPSOQ II (Pejtersen et al., 2010). Three items (e.g., “Do you feel part of a community at your place of work?”) were answered on a five-point scale (1 = *not at all*; 2 = *a small part of the time*; 3 = *part of the time*; 4 = *a large part of the time*; 5 = *all the time*). Higher values indicate more sense of social community.

#### Sleeping Troubles

Participants were asked about sleeping troubles with the subscale *Sleeping Troubles* from the COPSOQ II (Pejtersen et al., 2010). Four items (e.g., “How often have you slept badly and restlessly?”) were answered on a five-point scale (1 = *not at all*; 2 = *a small part of the time*; 3 = *part of the time*; 4 = *a large part of the time*; 5 = *all the time*). Higher values indicate more sleeping troubles.

#### Physical Health Impairment

We assessed participants self-reported physical health levels with the *SF-36 Version 2* (Jenkinson et al., 1999). Participants were asked to indicate their agreement to four items (e.g., “Because of your physical health, you were limited in the kind of work or other activities.”) on a scale from 1 = *none of the time* to 5 = *all of the time*. Higher values indicate worse physical health.

#### Work Productivity and Activity Impairment

The *Work Productivity and Activity Impairment Questionnaire: General Health V2.0* (WPAI:GH; Reilly et al., 1993) asked participants about the impact of health problems on their ability to work and perform regular activities over the past week. Three items (i.e., “During the past 7 days, how many hours did you miss from work because of your health problems?” “…, how many hours did you miss from work because of any other reason, such as vacation, holidays, time off to participate in this study?” “…, how many hours did you actually work?”) were provided with open text fields. Two items asked for a rating on a bipolar 11-point scale (e.g., “…, how much did your health problems affect your productivity while you were working?”; 0 = *Health problems had no effect on my work* to 10 = *Health problems completely prevented me from working*). This scale was not analyzed further as part of this research, but is reported for transparency reasons.

### Statistical Analyses

To obtain the final data set, we augmented the self-reported outcome measures at the four measurement points with the (self-reported) demographic information at baseline (i.e., age, gender), trial site, experimental group, and actual user behavior metrics from the app (i.e., number of daily sessions completed, number of nights tracked via the sleep tracker). Combining the self-reported DVs with predictors mirroring actual user behavior is a particular strength of the methodological approach of this study, as it eliminates the subjectivity in measuring compliance to the intervention by introducing an objective, fine-grained measure of app use intensity.

The data were analyzed by means of multilevel modeling for repeated measures where measurements (Level 1) are nested within subjects (Level 2). Compared to repeated measures ANOVAs, multilevel analyses possess the advantage of being more robust with respect to missing data and underlying variance/co-variance assumptions (Hox, 2010). Additionally, particularly in the case of the current project, multilevel analyses potentially allow the addition of “trial site” as Level 3 (i.e., measurement occasions within subjects within trial sites). The recommendation for each level is a minimum number of 30 cases per level (Hox, 2010). However, the study was conducted at only six trial sites. Thus, we could not include a third level into our analyses. Instead, the variable was disaggregated onto Level 2 (see controlled model), in order to assess the potential impact of higher order variables. The data were analyzed using the statistical programing language R (R Core Team, 2018) in combination with the *nlme* package (Pinheiro et al., 2018). Results were replicated with HLM 7 for Windows (Raudenbush et al., 2011).

Using the notation of Raudenbush et al. (2011), the foundational, uncontrolled Level 1 model can be written as follows: within subject *i*, the outcome measurement *OUTCOME*_*ti*_ at measurement time *t* depends on a baseline mean and a treatment effect according to the simple regression model,

$$O\,U\,T\,C\,O\,M\,E_{t\,i}=\pi_{0\,i}+\pi_{1\,i}\,T\,I\,M\,E_{t\,i}+e_{t\,i},$$

for subject *i*; *e*_*ti*_ is a measurement specific residual assumed to be independently and normally distributed within subjects. *TIME* takes the values 0, 1, and 2, so that the intercept denotes the baseline outcome.

Within the framework of the hierarchical linear model, the coefficients at Level 1 become outcome variables at Level 2. The intercept and subject specific treatment effect vary randomly across subjects (“random intercepts and slopes model”) according to the regression models,

$$\pi_{0\,i}=\beta_{00}+\beta_{01}\,G\,R\,O\,U\,P_{i}+r_{0\,i}$$

$$\pi_{1\,i}=\beta_{10}+\beta_{11}\,G\,R\,O\,U\,P_{i}+r_{1\,i}$$

Here, *β*_*00*_ is the grand mean for the baseline outcome and *β*_*10 *_is the average treatment effect (for one unit of *TIME*, i.e., per 2 weeks). *GROUP*_*i*_ is a dummy coded treatment contrast, with a value of 1 for the experimental group and 0 for the control group. *r*_*0i*_ and *r*_*1i*_ are subject specific random effects that are independent of *e*_*ti*_ and are assumed to have a bivariate normal distribution over subjects.

After establishing the basic treatment effect of the intervention (including T1, T2, and T3), the uncontrolled model was then extended in three distinct ways:

(a) Controlled model: potentially confounding factors including age (normalized to mean 0), gender (effect coded: male = −1, female = 1), trial site (effect coded: trial site 6 = −1) were introduced as additional predictors on Level 2,
(b) Intensity model: the categorical variable *GROUP*_*i*_ was substituted by the numerical measures of app use intensity: sessions completed (values 0–28) and nights tracked (values 0–28), and
(c) Follow-up model: the follow-up measurement T4 was included in a piecewise linear model.

## Results

### Basic Treatment Effect: Stress, Wellbeing, Resilience, and Sleeping Troubles Over Time

To test our hypotheses, we analyzed (1) between group differences, (2) changes over time, and (3) interaction effects of group and time. Results of the multilevel analyses are based on *n* = 532 participants (app group: *n* = 210; waitlist control group: *n* = 322) and measurement occasions T1 (baseline), T2 (mid-intervention), and T3 (post-intervention).^4^ Detailed results of the basic treatment effects are displayed in Table 3.

**TABLE 3:** Basic treatment model: longitudinal hierarchical linear models for fixed factor group (Level 2: app group vs. waitlist control) over time (Level 1).

	**General stress**	**Cognitive stress**	**Wellbeing**	**Resilience**	**Sleeping troubles**	**Social community**	**Physical health impairment**
**Fixed effects**							
Intercept B (SE)	3.01 (0.04)^∗∗∗^	2.62 (0.05)^∗∗∗^	3.24 (0.03)^∗∗∗^	4.97 (0.05)^∗∗∗^	2.85 (0.06)^∗∗∗^	3.87 (0.04)^∗∗∗^	2.05 (0.06)^∗∗∗^
Time slope B (SE)	−0.10 (0.02)^∗∗∗^	−0.08 (0.02)^∗∗∗^	0.05 (0.02)^∗∗∗^	0.06 (0.02)^∗∗^	−0.12 (0.02)^∗∗∗^	−0.01(0.02)	−0.04(0.03)
Group intercept B (SE)	−0.04(0.07)	−0.04(0.07)	0.02 (0.05)	−0.02(0.08)	−0.17(0.09)	−0.07(0.07)	−0.10(0.09)
Group^∗^Time slope B (SE)	−0.15 (0.04)^∗∗∗^	−0.11 (0.03)^∗∗^	0.08 (0.03)^∗∗^	0.03 (0.04)	−0.06(0.04)	0.05 (0.03)	0.06 (0.05)
**Random effects (variance components)**							
Intercept (SD)	0.39 (0.63)	0.51 (0.72)	0.27 (0.52)	0.68 (0.83)	0.77 (0.88)	0.45 (0.67)	0.74 (0.86)
Time slope (SD)	0.03 (0.18)	0.03 (0.16)	0.01 (0.08)	0.02 (0.16)	0.03 (0.17)	0.02 (0.16)	0.07 (0.26)
Level 1 error (SD)	0.17 (0.41)	0.14 (0.38)	0.11 (0.33)	0.17 (0.41)	0.24 (0.49)	0.12 (0.35)	0.33 (0.57)
Deviance (k)	2449.88 (8)	2392.21 (8)	1857.31 (8)	2640.29 (8)	2935.49 (8)	2198.30 (8)	3181.29 (8)

**N*_*Level*__1_ = 1261–1283, *N*_*Level*__2_ = 513–521; *k* = number of parameters in model. Time was coded continuously (T1 = 0, T2 = 1, T3 = 2). Group (dummy coded): Waitlist control = 0, App group = 1. Method of estimation: full maximum likelihood. The reported estimations are fixed effects with standard errors. Varying degrees of freedom due to selectively missing values. ^∗∗∗^*p* < 0.001, ^∗∗^*p* < 0.01, ^∗^*p* < 0.05.*

#### Stress

The results revealed an improvement in the experience of general stress and cognitive stress with a continuous time-trend toward experiencing less stress (significant time slopes), as well as a significant difference between the two groups over time (significant group^∗^time interactions). Persons in the app group experienced a greater decrease in both general and cognitive stress from T1 to T3 compared to the waitlist control group.

#### Wellbeing

Similarly, regarding wellbeing, there was a significant time-trend toward reporting more wellbeing over time (significant time slope). Participants in the app group compared to the waitlist control group reported significantly more wellbeing over time (significant group^∗^time interaction).

#### Resilience

We could not confirm our hypothesis with respect to resilience. While there was indeed a significant time-trend toward reporting more resilience over time (significant time slope), the difference between the two groups over time was not significant (group^∗^time interaction n.s.).

#### Sleeping Troubles

Self-reported sleeping troubles improved over time with a significant trend toward reporting reduced sleeping troubles (significant time slope). The difference between the two groups was not significant, even though descriptively, the app group showed a larger improvement (group^∗^time interaction n.s.).

### Intensity Model: Engagement With the App as a Predictor

We conducted a second set of analyses to examine whether app use intensity had an influence on changes in the DVs over time. We hypothesized that the more interaction with the app occurs over time, the larger the improvement. Specifically, we examined whether the number of completed sessions (see Table 4) and the number of tracked nights (see Supplementary Material for details) on the app predicted changes in the DVs over time. On average, participants in the app group completed *M* = 11.06 sessions (*SD* = 7.34, range: 1–28) and tracked *M* = 3.61 nights (*SD* = 6.11, range: 0–25). A substantial number of people in the app group (*n* = 111, 52.9%) did not track their sleep at all, and *n* = 22 (10.5%) only tried once.

**TABLE 4:** Intensity model: longitudinal hierarchical linear models for fixed factor number of sessions completed (Level 2: user metrics) over time (Level 1).

	**General stress**	**Cognitive stress**	**Wellbeing**	**Resilience**	**Sleeping troubles**	**Social community**	**Physical health impairment**
**Fixed effects**							
Intercept B (SE)	2.99 (0.04)^∗∗∗^	2.60 (0.04)^∗∗∗^	3.27 (0.03)^∗∗∗^	5.00 (0.05)^∗∗∗^	2.82 (0.05)^∗∗∗^	3.87 (0.04)^∗∗∗^	2.02 (0.05)^∗∗∗^
Time slope B (SE)	−0.11 (0.02)^∗∗∗^	−0.09 (0.02)^∗∗∗^	0.05 (0.01)^∗∗^	0.05 (0.02)^∗^	−0.12 (0.02)^∗∗∗^	−0.00(0.02)	−0.02(0.03)
Sessions intercept B (SE)	−0.00(0.00)	0.00 (0.00)	−0.00(0.00)	−0.01(0.01)	−0.01(0.01)	−0.01(0.00)	−0.00(0.01)
Sessions^∗^Time slope B (SE)	−0.01 (0.00)^∗∗∗^	−0.01 (0.00)^∗∗∗^	0.01 (0.00)^∗∗∗^	0.00 (0.00)^∗^	−0.00 (0.00)	0.00 (0.00)	0.00 (0.00)
**Random effects (variance components)**							
Intercept (SD)	0.39 (0.63)	0.51 (0.72)	0.27 (0.52)	0.68 (0.83)	0.78 (0.88)	0.45 (0.67)	0.74 (0.86)
Time slope (SD)	0.03 (0.18)	0.03 (0.16)	0.01 (0.07)	0.02 (0.15)	0.03 (0.17)	0.02 (0.16)	0.07 (0.26)
Level 1 error (SD)	0.17 (0.41)	0.14 (0.38)	0.11 (0.33)	0.17 (0.41)	0.24 (0.49)	0.12 (0.35)	0.33 (0.57)
Deviance (k)	2452.20 (8)	2392.30 (8)	1851.13 (8)	2636.20 (8)	2935.80 (8)	2198.37 (8)	3182.66 (8)

**N*_*Level*__1_ = 1261–1283, *N*_*Level*__2_ = 513–521; *k* = number of parameters in model. Time was coded continuously (T1 = 0, T2 = 1, T3 = 2). Method of estimation: full maximum likelihood. The reported estimations are fixed effects with standard errors. Varying degrees of freedom due to selectively missing values. ^∗∗∗^*p* < 0.001, ^∗∗^*p* < 0.01, ^∗^*p* < 0.05.*

Confirming Hypothesis 2, the results revealed that the more sessions users completed, the less general and cognitive stress they reported over time (significant sessions^∗^time interactions). The same applied to self-reported wellbeing: the more sessions users completed, the larger their increase in wellbeing over time (significant sessions^∗^time interaction). In case of resilience, the interaction effect of sessions^∗^time also became significant, while it had only been trending when experimental group was the predictor (see basic treatment effects).

Regarding sleeping troubles, the number of completed sessions was only a trend-significant predictor for an improvement, yet descriptively the results pointed in the expected direction. As the app offers the option to track sleep, and thus, focus specifically on creating awareness for and improving this health-related issue, we included the number of tracked nights instead of sessions as a predictor for sleeping troubles. Number of tracked nights turned out to be a significant predictor for the improvement in sleeping troubles over time (significant nights^∗^time interaction, *p* < 0.001; see Supplementary Material for details).

### Social Community at Work and Physical Health Impairment

Regarding the first open research question, the results revealed neither a significant time trend nor an intervention effect concerning participants’ perception of their social community at work. The physical health impairment scale was included as a measure that should be insensitive to the mental health intervention. Indeed, we did not observe a change in physical health over time and no significant difference between the two groups. Social community at work and physical health impairment were also not affected by app use intensity (i.e., sessions completed or nights tracked). This suggests that only aspects of persons’ mental health but not their physical health or perception of organizational climate were directly influenced by using the app.

### Follow-Up Model: Effects of App Use at Follow-Up

**FIGURE 1:**
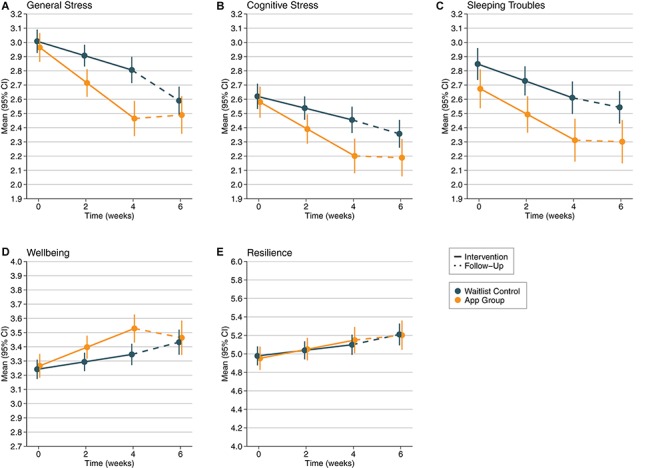
Development of **(A)** General Stress (scale 1–5), **(B)** Cognitive Stress (scale 1–5), **(C)** Sleeping Troubles (scale 1–5), **(D)** Wellbeing (scale 1–5), and **(E)** Resilience (scale 1–7) over time from T1 to T4 (intervention phase from T1 to T3, follow-up from T3 to T4) depending on experimental group (app group vs. waitlist control). Error bars represent 95% confidence limits.

Our second open research question aimed at examining the sustainment of effects after people stopped using the app in comparison to the waitlist control group. Thus, in a third step, we included the 2-week follow-up measurement occasion (T4) into our analyses. To analyze a potentially non-linear continuation into the follow-up trend, we incorporated two differently coded time predictors into the hierarchical analyses (time1: 0-1-2-2; time2: 0-0-0-1); additionally, group and the interaction terms (time1^∗^group and time2^∗^group) served as predictors for the DVs. The results revealed a significant interaction of group and time2 regarding general stress (*beta* = 0.24, *SE* = 0.07, *p* < 0.001) and wellbeing (*beta* = −0.15, *SE* = 0.07, *p* = 0.02), but not regarding any of the other DVs (all *p*s > 0.18). Remarkably, these findings indicate that while the effects remained stable in the app group, the control group significantly improved from T3 to T4 (see Figure 1 and see section “Discussion”).

## Discussion

Systematic reviews and meta-analyses show that there are only few scientifically validated mobile health interventions on the market, and even fewer applications designed for workplace interventions, which look at both individual mental health and overall organizational culture change (e.g., Zhao et al., 2016; Stratton et al., 2017; McKay et al., 2018). This RCT adds to the sparse research on preventative mental health solutions in the workplace. It was conducted in a ‘naturalistic environment’, as it was implemented in different work environments, in three countries, and across disciplines. By executing the trial as an experimental field study, and as such, including a large variety of workplaces and people, the current research bears high external validity. Our results are in line with recent research findings, suggesting that digital health interventions can improve mental health-related outcomes in a work context (e.g., Tan et al., 2014; Mistretta et al., 2018). Yet, they go beyond previous studies, as this large-scale RCT included multiple workplaces and addressed both, positive mental health of individuals and indicators of organizational change. In addition to the core findings of this study, we also discuss aspects of user attrition and sustained engagement as important practical issues to address (cf. Howarth et al., 2018; see section “Practical implications”).

Regarding individual mental health, the app was shown to be effective relative to a waitlist control group. Supporting our hypotheses, the results of this RCT indicate that using the app improves indicators of stress and wellbeing, and also, to a lesser extent, resilience. The results further suggest that within the app group, the positive effects remained stable after a period of 2 weeks without app usage. As indicated above, resilience comprises both, a state and a trait component (cf. Windle, 2010). Due to the fact that the resilience measure used in the current research also incorporates trait-like aspects, improvements in resilience likely require more time to develop. Job resources, as defined in the JDR (Demerouti et al., 2001), can take more time to accrue than 4 weeks to realize their full potential in stimulating personal growth. The actual content that users chose on the app^5^ (i.e., the goals and sessions they self-selected and completed within the interventional module), may further explain the specific effects on indicators of individual mental health outcomes (i.e., stress, wellbeing, resilience). Overall, we found the app to be effective to improve stress and wellbeing outcomes of all employees, regardless of their age, gender, and workplace.

To observe change processes in the organizations, we also examined the outcome variable ‘social community at work’ (i.e., a measure of the organizational climate). Based on anecdotal evidence from users in previous trials, we assumed that undergoing a joint wellbeing intervention may create some “common ground” among employees and increase group cohesiveness (Mistretta et al., 2018). Yet, no such effects manifested on the respective scale. However, we observed a significant improvement in general stress and wellbeing within the waitlist control group from T3 to T4. Since these improvements are confined to those outcome variables that responded to the intervention from T1-T3 (with cognitive stress trending into a similar direction), but did not materialize on the social community and physical health impairment scales, we suggest that it is unlikely that these changes took place only because of measurement effects. They appear to be neither random nor spontaneous. While we cannot provide a perfect explanation for this effect, we argue that effective aspects of the intervention may have spread across the organization after the active treatment phase had ended, potentially due to communication processes within the organizations. Previous research has shown that positive behavior changes can be contagious, for example, by communicating social norms (Goldstein et al., 2008). The current results are a first indicator that offering a digital health solution in the workplace might be beneficial not only for those who decide to make use of it, but also for their colleagues. Certainly, further research is needed to examine these potential carry-over effects over a longer time period.

The “Kelaa” app meets the criteria that have been recommended by previous researchers: It is grounded in theory (i.e., the JDR*;* Bakker and Demerouti, 2007), offers a variety of evidence-based interventions that target specific challenges that employees might face in the work context, and implements various science-backed behavior change strategies. Our findings indicate that more intense use of the interventional module of the app increased the beneficial effects on stress and wellbeing, while specifically using the sleep tracking functionality can help to reduce sleeping troubles. This highlights the impact of self-monitoring and connects the study to a body of medical literature documenting diary-based approaches to tackle sleep impairment (Carney et al., 2012), in particular in the context of mHealth (Lorenz and Williams, 2017). Taken together, the current results support the assumption that building mobile health interventions on a theoretical foundation and using scientific methods bears great potential to positively influence mental health-related outcomes (cf. Webb et al., 2010; Donker et al., 2013; Bolier and Abello, 2014).

### Practical Implications

Attrition in digital interventions is a challenge. Attrition rates can spike with up to 64% in motivated, self-selected groups (Howells et al., 2016); however, they are usually lower in regular working samples (Payne et al., 2015; Howarth et al., 2018). This is a known barrier to workplace interventions. Developers of digital mental health interventions have been recommended to implement a behavior change plan and an interactive framework to increase user engagement (Bakker et al., 2016). The Kelaa Mental Resilience App was developed based on these recommendations. Yet, we also observed moderate to high rates of non-adherence to the trial participation guidelines, in particular in the intervention group. We acknowledge that app-based interventions might not be suitable for everyone, which might have resulted in a self-selection mechanism over time. Broadening the scope of the content within the app while keeping the personalized approach for each individual might help to make it more applicable for a larger variety of users’ needs.

Further, the relatively low compliance in questionnaire completion and variance of completion rates between trial sites suggests that company-specific communication strategies may play a key role in user engagement. Strategies to make using the mobile health intervention a more social experience, and thus, increasing the sense of social community might be beneficial in intensifying intervention impact (e.g., van Dick and Haslam, 2012). Prevention programs can be targeted universally across an organization, to individuals at high risk, or to individuals who show initial symptoms of risk (Cuijpers et al., 2012). Creating and implementing structured, targeted psychological interventions is more likely to be effective than generic stress management trainings (Ebert et al., 2016). In line with previous research, we suggest that interventions should be targeted at both the individual and the organization, as the inclusion of team-based interventions can improve workplace stress (Tan et al., 2014). In general, the best outcomes may be achieved by providing the right type of intervention to the respective population, as this fit will likely influence the results (see Stratton et al., 2017).

### Limitations and Future Research Directions

Despite our contribution to both research and practice, some limitations need to be noted. First, our *a priori* sample size calculation indicated that we would have needed more participants (*N* = 561 vs. *n* = 532). Further, we acknowledge the possibility of false positives due to multiple hypotheses testing. However, the effects that we found were large enough to be detected with the given sample size. Therefore, we conclude that the changes in the DVs, which originated from using the app, were large enough to be meaningful for employees’ experiences of stress and wellbeing.

Second, the sample was rather heterogeneous, as described above. The over-representation of female participants as well as the relatively high educational standard in the sample is a reflection of the respective companies that participated in the trial. Despite the statistical precautions that were taken (see controlled model in the online Supplementary Material), the findings may not apply to other workplace populations. We recommend being cautious with over-generalizations of the findings.

Third, the scales used to assess the DVs varied in terms of their sensitivity to change. Thus, some may not have been sensitive enough to capture smaller changes that occurred, such as fluctuations in mood. For instance, the scale that was used to assess resilience targets rather stable (trait-like) features, while the scales for stress and wellbeing are more sensitive to short-term changes (states). This might have contributed to the less clear-cut effects for resilience. In addition, although previous studies have also shown a positive impact in stress reduction, these may not necessarily lead to an impact on work-related outcomes such as absenteeism (Joyce et al., 2016). More research in this area is needed.

Fourth, we did not conduct the study at enough trial sites, and we could not recruit sufficient participants within each site to include organization as a third level into our multilevel analyses. Thus, the questions remain whether we are able to generalize the findings across trial sites and whether it was more effective at certain sites compared to others. The results of the controlled model suggest that the treatment effect was present at all sites, regardless of differences in employees’ stress and wellbeing baselines (see Supplementary Material). However, this finding should not be overgeneralized. Working with larger datasets by including more trial sites and associated context variables would allow a data-driven tailoring of the mobile health intervention to fit the individual needs of each organization and its employees.

Fifth, due to the personalization, and thus, the large variability in how people used the app, regarding both the tracking and the interventional module, we cannot conclude which of the elements contributed to the app being effective in reducing stress and increasing wellbeing. In order to identify the best combination of interventions and the most effective elements of the app, more data points would be needed. Analyses with larger data sets, which include detailed information on user metrics, could lead to more efficient interventions. To gain more differentiated insight, we support the call for better ways to assess the effectiveness of apps for health behavior change (McKay et al., 2018).

Last, due to the naturalistic nature of the RCT, there was a range of factors that could not be controlled for, and which therefore may have influenced the DVs over and above the intervention. Thus, at the cost of the high external validity, our study has rather low internal validity. While participants in both treatment and control group were instructed not to discuss contents of the study with members of the other group, it is difficult to enforce this constraint in practice. Both in the controlled and uncontrolled model, we witnessed significant effects of time, irrespective of experimental group, for stress, wellbeing, resilience, and sleeping troubles (significant time slopes). These effects were consistently smaller than the treatment effects, yet noteworthy. We suspect that they may result from perceived changes to the organizational context due to the sheer existence of the intervention. Recruitment efforts within the organizations, which included advertisements for an ‘innovation and research project’, likely have signaling effects for employees to notice that the employer is taking employee wellbeing seriously. Despite these limitations, the current findings suggest that there is a benefit to using technological solutions to mental health in the workplace to support organizations and their employees to thrive.

## Conclusion

This paper presents conclusive evidence that a smartphone-based health intervention that is grounded in the JDR (Bakker and Demerouti, 2007) can decrease levels of perceived stress and sleeping troubles, and improve subjective wellbeing and resilience after 4 weeks, with sustained results at a 6-week follow-up. To the best of our knowledge, the current project offers the first large-scale, multi-center RCT using a theoretically sound smartphone application to manage and reduce stress in a work context, based on a sample of employees at various companies. The study therefore contributes to closing the empirical evidence gap concerning the effectiveness of app-based interventions to manage stress and positive mental health at work, in order to fulfill their potential as “enabler[s] of change” (Stevenson and Farmer, 2017, p. 7). While mental health professionals in traditional health care have an ethical obligation to provide patients with theoretically and empirically sound interventions (e.g., American Psychological Association, 2002; American Counseling Association, 2014), we argue that organizations should do the same for their employees. To reduce the costs of ill-health and keep organizations and their employees thriving, more research on effective solutions to positive mental health in the workplace is needed.

## Data Availability Statement

The raw data supporting the conclusions of this article will be made available by the authors, without undue reservation, to any qualified researcher.

## Ethics Statement

Ethics approval was provided by an Independent Ethics Advisory Board (EAB) according to the European Commission Horizon 2020 Ethics Appraisal Procedure (European Commission, 2018), consisting of four independent ethics advisors from Germany and the United Kingdom. All members of the EAB either signaled “favorable opinion” or “favorable opinion with additional conditions.” Ethics approval was required and obtained as per applicable institutional and national guidelines and regulations. Data protection policies according to GDPR guidelines were strictly followed. Participation in the study was voluntary. All participants gave their informed consent in written form.

## Author Contributions

SW conceptualized and designed the RCT and wrote the manuscript with valuable input from CL and NH. CL and NH implemented the study. CL organized the database and created the figures. SW and CL carried out the statistical analyses, interpreted the results, and created the tables. NH conducted a systematic literature search. All authors contributed to the manuscript revision, read, and approved the submitted version.

## Conflict of Interest

SW was an independent research consultant with Soma Analytics/DNAFit, the developers and providers of the app. As a freelancer, she was compensated for her work on this research by Soma Analytics/DNAFit as part of the European Union’s Horizon 2020 project. CL is a co-founder of and was an employee with Soma Analytics/DNAFit, the developers and providers of the app. NH was an employee with Soma Analytics, the developers and providers of the app.

## Footnotes

1 *Positive mental health* goes beyond the absence of psychopathological symptoms. It can be defined as the subjective experience of wellbeing and an optimal way of psychological and social functioning (cf. World Health Organization [WHO], 2018).

2 *Mental ill-health* (or mental health problems) is broadly defined as including established diagnoses (e.g., depression, anxiety disorders, burnout) and work-related stress symptoms (cf. Harvey et al., 2017).

3 *mHealth* is defined as “medical and public health practice supported by mobile devices, such as mobile phones, patient monitoring devices, personal digital assistants (PDAs), and other wireless devices” (World Health Organization [WHO], 2011, p. 6).

4 Controlled model (see Supplementary Material): All analyses were rerun with age, gender, and trial site as additional predictors (entered on Level 2). None of the basic treatment effects changed when we included the control variables. For the extended models, sample sizes may differ due to selectively missing data as indicated in the respective tables.

5 User metrics revealed the popularity of each goal (see Table 1).

## References

[B1] American Counseling Association (2014). *Code of Ethics.* Available at: https://www.counseling.org/resources/aca-code-of-ethics.pdf (accessed September 14, 2017).

[B2] American Psychological Association (2002). Ethical principles of psychologists and code of conduct. *Am. Psychol.* 57 1060–1073. 10.1037//0003-066X.57.12.1060 12613157

[B3] Aryana B. Brewster L. Nocera J. A. (2018). Design for mobile mental health: an exploratory review. *Health Technol.* 9 401–424. 10.1007/s12553-018-0271-1 20439251

[B4] Bakker A. B. Demerouti E. (2007). The job demands-resources model: state of the art. *J. Manag. Psychol.* 22 309–328. 10.1108/02683940710733115

[B5] Bakker A. B. Demerouti E. Schaufeli W. B. (2003). Dual processes at work in a call centre: an application of the job demands–resources model. *Eur. J. Work Organ. Psychol.* 12 393–417. 10.1080/13594320344000165

[B6] Bakker D. Kazantzis N. Rickwood D. Rickard N. (2016). Mental health smartphone apps: review and evidence-based recommendations for future developments. *JMIR Ment. Health* 3 1–31. 10.2196/mental.4984 26932350 PMC4795320

[B7] Barak A. Hen L. Boniel-Nissim M. Shapira N. A. (2008). A comprehensive review and a meta-analysis of the effectiveness of internet-based psychotherapeutic interventions. *J. Technol. Hum. Serv.* 26 109–160. 10.1080/15228830802094429

[B8] Birney A. J. Gunn R. Russell J. K. Ary D. V. (2016). MoodHacker mobile web app with email for adults to self-manage mild-to-moderate depression: randomized controlled trial. *JMIR Mhealth Uhealth* 4 1–19. 10.2196/mhealth.4231 26813737 PMC4748138

[B9] Bolier L. Abello K. M. (2014). “Online positive psychological interventions: state of the art and future directions,” in *The Wiley Blackwell Handbook of Positive Psychological Interventions*, 1st Edn, eds Schueller S. Parks A. C. (Chichester: Wiley-Blackwell), 286–309.

[B10] Broughton A. (2010). *Work-Related Stress.* Available at: https://www.eurofound.europa.eu/observatories/eurwork/comparative-information/work-related-stress (accessed June 10, 2017).

[B11] Carlbring P. Andersson G. Cuijpers P. Riper H. Hedman-Lagerlöf E. (2018). Internet-based vs. face-to-face cognitive behavior therapy for psychiatric and somatic disorders: an updated systematic review and meta-analysis. *Cogn. Behav. Ther.* 47 1–18. 10.1080/16506073.2017.1401115 29215315

[B12] Carney C. E. Buysse D. J. Ancoli-Israel S. Edinger J. D. Krystal A. D. Lichstein K. L. (2012). The consensus sleep diary: standardizing prospective sleep self-monitoring. *Sleep* 35 287–302. 10.5665/sleep.1642 22294820 PMC3250369

[B13] Chandola T. Brunner E. Marmot M. (2006). Chronic stress at work and the metabolic syndrome: prospective study. *BMJ* 332 521–525. 10.1136/bmj.38693.435301.80 16428252 PMC1388129

[B14] Connor K. M. Davidson J. R. T. (2003). Development of a new resilience scale: the connor-davidson resilience scale (CD-RISC). *Depress. Anxiety* 18 76–82. 10.1002/da.10113 12964174

[B15] Cuijpers P. Beekman A. T. Reynolds C. F. (2012). Preventing depression: a global priority. *JAMA* 307 1033–1034. 10.1001/jama.2012.271 22416097 PMC3397158

[B16] Deady M. Johnston D. A. Glozier N. Milne D. Choi I. Mackinnon A. (2018). A smartphone application for treating depressive symptoms: study protocol for a randomised controlled trial. *BMC Psychiatry* 18:166. 10.1186/s12888-018-1752-5 29859060 PMC5984798

[B17] Deloitte (2017). *Mental Health and Employers: The Case for Investment. Supporting Study for the Independent Review.* Available at: https://www2.deloitte.com/content/dam/Deloitte/uk/Documents/public-sector/deloitte-uk-mental-health-employers-monitor-deloitte-oct-2017.pdf (accessed September 18, 2018).

[B18] Demerouti E. Bakker A. B. Nachreiner F. Schaufeli W. B. (2001). The job demands-resources model of burnout. *J. Appl. Psychol.* 86 499–512. 10.1037/0021-9010.86.3.499 11419809

[B19] Donker T. Petrie K. Proudfoot J. Clarke J. Birch M. R. Christensen H. (2013). Smartphones for smarter delivery of mental health programs: a systematic review. *J. Med. Internet Res.* 15 1–32. 10.2196/jmir.2791 24240579 PMC3841358

[B20] Ebert D. D. Kählke F. Buntrock C. Berking M. Smit F. Heber E. (2016). A health economic outcome evaluation of an internet-based mobile-supported stress management intervention for employees. *Scand. J. Work Environ. Health* 44 171–182. 10.5271/sjweh.3691 29144535

[B21] Ebert D. D. Lehr D. Smit F. Zarski A. C. Riper H. Heber E. (2014). Efficacy and cost-effectiveness of minimal guided and unguided internet-based mobile supported stress-management in employees with occupational stress: a three-armed randomised controlled trial. *BMC Public Health* 14:807 10.1186/1471-2458-14-807 PMC4153891 25099533

[B22] European Agency for Safety and Health at Work (2014). *Calculating the cost of work-related stress and psychosocial risks: European Risk Observatory Literature Review.* Available at: https://osha.europa.eu/en/tools-and-publications/publications/literature_reviews/calculating-the-cost-of-work-related-stress-and-psychosocial-risks (accessed September 18, 2018).

[B23] European Commission (2018). *Horizon 2020 Ethics Appraisal Procedure.* Available at: http://ec.europa.eu/research/participants/docs/h2020-funding-guide/cross-cutting-issues/ethics_en.htm (accessed January 12, 2018).

[B24] Faul F. Erdfelder E. Lang A.-G. Buchner A. (2007). G^∗^Power 3: a flexible statistical power analysis program for the social, behavioral, and biomedical sciences. *Behav. Res. Methods* 39 175–191. 10.3758/BF03193146 17695343

[B25] Fergus S. Zimmerman M. A. (2005). Adolescent resilience: a framework for understanding healthy development in the face of risk. *Annu. Rev. Public Health* 26 399–419. 10.1146/annurev.publhealth.26.021304.144357 15760295

[B26] Firth J. Torous J. Nicholas J. Carney R. Pratap A. Rosenbaum S. (2017). The efficacy of smartphone-based mental health interventions for depressive symptoms: a meta-analysis of randomized controlled trials. *World Psychiatry* 16 287–298. 10.1002/wps.20472 28941113 PMC5608852

[B27] Gaggioli A. Riva G. (2013). From mobile mental health to mobile wellbeing: opportunities and challenges. *Stud. Health Technol. Inform.* 184 141–147. 10.3233/978-1-61499-209-7-141 23400146

[B28] Goldstein N. J. Cialdini R. B. Griskevicius V. (2008). A room with a viewpoint: using social norms to motivate environmental conservation in hotels. *J. Consum. Res.* 35 472–482. 10.1086/586910

[B29] Hakanen J. J. Schaufeli W. B. Ahola K. (2008). The job demands-resources model: a three-year cross-lagged study of burnout, depression, commitment, and work engagement. *Work Stress* 22 224–241. 10.1080/02678370802379432

[B30] Hammen C. (2005). Stress and depression. *Annu. Rev. Clin. Psychol.* 1 293–319. 10.1146/annurev.clinpsy.1.102803.143938 17716090

[B31] Harvey S. B. Modini M. Joyce S. Milligan-Saville J. S. Tan L. Mykletun A. (2017). Can work make you mentally ill? A systematic meta-review of work-related risk factors for common mental health problems. *Occup. Environ. Med.* 74 301–310. 10.1136/oemed2016-104015 28108676

[B32] He Y. Li Y. (2013). Physical activity recognition utilizing the built-in kinematic sensors of a smartphone. *Int. J. Distrib. Sens. Netw.* 9 1–11. 10.1155/2013/481580

[B33] Health and Safety Executive (2017). *Tackling Work-Related Stress Using the Management Standards Approach: A Step-by-Step Workbook.* Available at: http://www.hse.gov.uk/pubns/wbk01.pdf (accessed September 18, 2018).

[B34] Heber E. Lehr D. Ebert D. D. Berking M. Riper H. (2016). Web-based and mobile stress management intervention for employees: a randomized controlled trial. *J. Med. Internet Res.* 18:e21 10.2196/jmir.5112 PMC4749847 26818683

[B35] Howarth A. Quesada J. Silva J. Judycki S. Mills P. R. (2018). The impact of digital health interventions on health-related outcomes in the workplace: a systematic review. *Digital Health* 4 1–18. 10.1177/2055207618770861 29942631 PMC6016571

[B36] Howells A. Ivtzan I. Eiroa-Orosa F. J. (2016). Putting the “app” in happiness: a randomised controlled trial of a smartphone-based mindfulness intervention to enhance wellbeing. *J. Happiness Stud.* 17 163–185. 10.1007/s10902-014-9589-1

[B37] Hox J. J. (2010). *Multilevel Analysis: Techniques and Applications*, 2nd Edn, New York, NY: Routledge.

[B38] Jenkinson C. Stewart-Brown S. Petersen S. Paice C. (1999). Assessment of the SF-36 version 2 in the United Kingdom. *J. Epidemiol. Commun. Health* 53 46–50. 10.1136/jech.53.1.46 PMC1756775 10326053

[B39] Joyce S. Modini M. Christensen H. Mykletun A. Bryant R. Mitchell P. B. (2016). Workplace interventions for common mental disorders: a systematic meta-review. *Psychol. Med.* 46 683–697. 10.1017/S0033291715002408 26620157

[B40] Kompier M. (2002). The psychosocial work environment and health - what do we know and where should we go? *Scand. J. Work Environ. Health* 28 1–4. 10.5271/sjweh.639 11871847

[B41] Landsbergis P. A. (2003). The changing organization of work and the safety and health of working people: a commentary. *J. Occup. Environ. Med.* 45 61–72. 10.1097/00043764-200301000-00014 12553180

[B42] Larsen M. E. Huckvale K. Nicholas J. Torous J. Birrell L. Li E. (2019). Using science to sell apps: evaluation of mental health app store quality claims. *NPJ Digit. Med.* 2:18. 10.1038/s41746-019-0093-1 31304366 PMC6550255

[B43] Leppert K. Koch B. Brähler E. Strauß B. (2008). Die Resilienzskala (RS) – Überprüfung der Langform RS-25 und einer Kurzform RS-13. *Klin. Diagn. Eval.* 1 226–243.

[B44] Lorenz C. P. Williams A. J. (2017). Sleep apps: what role do they play in clinical medicine? *Curr. Opin. Pulm. Med.* 23 512–516. 10.1097/MCP.0000000000000425 28820754

[B45] Luthar S. S. Cicchetti D. (2000). The construct of resilience: implications for interventions and social policies. *Dev. Psychopathol.* 12 857–885. 10.1017/S0954579400004156 11202047 PMC1903337

[B46] Ly K. H. Asplund K. Andersson G. (2014). Stress management for middle managers via an acceptance and commitment-based smartphone application: a randomized controlled trial. *Internet Interv.* 1 95–101. 10.1016/j.invent.2014.06.003

[B47] Martinez R. Williams C. (2010). “Matching clients to CBT self-help resources,” in *Oxford Guide to Low intensity CBT Interventions*, eds Bennet-Levy J. Richards D. A. Farrand P. Christensen H. Griffiths K. M. Kavanaugh D. J. (Oxford: Oxford University Press), 113–121. 10.1093/med:psych/9780199590117.003.0009

[B48] McKay F. H. Cheng C. Wright A. Shill J. Stephens H. Uccellini M. (2018). Evaluating mobile phone applications for health behaviour change: a systematic review. *J. Telemed. Telecare* 24 22–30. 10.1177/1357633X16673538 27760883

[B49] Meyer M. Wenzel J. Schenkel A. (2018). *Krankheitsbedingte Fehlzeiten in der Deutschen Wirtschaft im Jahr 2017.* Available at: https://www.wido.de/fileadmin/Dateien/Dokumente/Publikationen_Produkte/Buchreihen/Fehlzeitenreport/wido_pra_fzr_2018_krankheitsbedingte_fehlzeiten.pdf (accessed January 15, 2019).

[B50] Mistretta E. G. Davis M. C. Temkit M. H. Lorenz C. Darby B. Stonnington C. M. (2018). Resilience training for work-related stress among health care workers: results of a randomized clinical trial comparing in-person and smartphone-delivered interventions. *J. Occup. Environ. Med.* 60 559–568. 10.1097/JOM.0000000000001285 29370014

[B51] Muaremi A. Arnrich B. Tröster G. (2013). Towards measuring stress with smartphones and wearable devices during workday and sleep. *BioNanoScience* 3 172–183. 10.1007/s12668-013-0089-2 25530929 PMC4269214

[B52] Nicholas J. Larsen M. E. E. Christensen H. Proudfoot J. (2015). Mobile apps for bipolar disorder: a systematic review of features and content validity. *J. Med. Internet Res.* 17 198–205. 10.2196/jmir.4581 26283290 PMC4642376

[B53] Ong A. D. Bergeman C. S. Bisconti T. L. Wallace K. A. (2006). Psychological resilience, positive emotions, and successful adaptation to stress in later life. *J. Personal. Soc. Psychol.* 91 730–749. 10.1037/0022-3514.91.4.730 17014296

[B54] Payne H. E. Lister C. West J. H. Bernhardt J. M. (2015). Behavioral functionality of mobile apps in health interventions: a systematic review of the literature. *JMIR Mhealth Uhealth* 3:e20. 10.2196/mhealth.3335 25803705 PMC4376122

[B55] Pejtersen J. H. Kristensen T. S. Borg V. Bjorner J. B. (2010). The second version of the copenhagen psychosocial questionnaire. *Scand. J. Public Health* 38 (3 Suppl.), 8–24. 10.1177/1403494809349858 21172767

[B56] Pinheiro J. Bates D. DebRoy S. Sarkar D. R Core Team, (2018). *nlme: Linear and Nonlinear Mixed Effects Models R package (Version 3.1-137) [Computer Software].* Available from https://CRAN.R-project.org/package=nlme (accessed August 24, 2018).

[B57] R Core Team (2018). *R: A Language and Environment for Statistical Computing [Computer Software].* Vienna: R Foundation for Statistical Computing.

[B58] Ranjbartabar H. Maddah A. Kavakli M. (2016). An eMental-health application and customised questionnaires for stress measurement. *Int. J. Comput. Inform. Sci.* 17 42–48.

[B59] Raudenbush S. W. Bryk A. S. Cheong Y. F. Congdon R. T. du Toit M. (2011). *HLM 7 for Windows [Computer Software].* Lincolnwood, IL: Scientific Software International Inc.

[B60] Reilly M. C. Zbrozek A. S. Dukes E. (1993). The validity and reproducibility of a work productivity and activity impairment measure. *Pharmacoeconomics* 4 353–365. 10.2165/00019053-199304050-00006 10146874

[B61] Research 2 Guidance (2017). *Mhealth app Economics 2017/2018: Current Status and Future Trends in Mobile Health.* Available at: https://research2guidance.com/325000-mobile-health-apps-available-in-2017/ (accessed January 15, 2019).

[B62] Schaufeli W. B. Bakker A. B. (2004). Job demands, job resources, and their relationship with burnout and engagement: a multi-sample study. *J. Organ. Behav.* 25 293–315. 10.1002/job.248

[B63] Schaufeli W. B. Taris T. W. (2014). “A critical review of the job demands-resources model: implications for improving work and health,” in *Bridging Occupational, Organizational and Public Health: A Transdisciplinary Approach*, eds Bauer G. F. Hämmig O. (Dordrecht: Springer), 43–68. 10.1007/978-94-007-5640-3_4

[B64] Schumacher J. Leppert K. Gunzelmann T. Strauß B. Brähler E. (2005). Die Resilienzskala – Ein Fragebogen zur Erfassung der psychischen Widerstandsfähigkeit als Personmerkmal. *Z. Klin. Psychol. Psychiatr. Psychother.* 53 16–39.

[B65] Sheldon K. M. Boehm J. Lyubomirsky S. (2013). “Variety is the spice of happiness: the hedonic adaptation prevention model,” in *Oxford Handbook of Happiness*, eds Boniwell I. David S. Conley Ayers A. (Oxford: Oxford University Press), 901–914.

[B66] Statista (2018). *Number of mHealth apps Available in the Apple App Store from 2nd Quarter 2015 to 3rd Quarter 2018.* Available at: https://www.statista.com/statistics/779910/health-apps-available-ios-worldwide/ (accessed January 15, 2019).

[B67] Stevenson D. Farmer P. (2017). *Thriving at Work. The Stevenson/Farmer Review of Mental Health and Employers.* Available at: https://assets.publishing.service.gov.uk/government/uploads/system/uploads/attachment_data/file/658145/thriving-at-work-stevenson-farmer-review.pdf (accessed September 18, 2018).

[B68] Stratton E. Lampit A. Choi I. Calvo R. A. Harvey S. B. Glozier N. (2017). Effectiveness of eHealth interventions for reducing mental health conditions in employees: a systematic review and meta-analysis. *PLoS One* 12:e0189904. 10.1371/journal.pone.0189904 29267334 PMC5739441

[B69] Tan L. Wang M. J. Modini M. Joyce S. Mykletun A. Christensen H. (2014). Preventing the development of depression at work: a systematic review and meta-analysis of universal interventions in the workplace. *BMC Med.* 12:74. 10.1186/1741-7015-12-74 24886246 PMC4014627

[B70] Tennant R. Hiller L. Fishwick R. Platt S. Joseph S. Weich S. (2007). The Warwick-Edinburgh mental well-being scale (WEMWBS): development and UK validation. *Health Qual. Life Outcomes* 5:63. 10.1186/1477-7525-5-63 18042300 PMC2222612

[B71] van Dick R. Haslam S. A. (2012). “Stress and well-being in the workplace: support for key propositions from the social identity approach,” in *The Social Cure: Identity, Health and Well-Being*, eds Jetten J. Haslam C. Haslam S. A. (New York, NY: Psychology Press), 175–194.

[B72] Wagnild G. M. Young H. M. (1993). Development and psychometric evaluation of the Resilience scale. *J. Nurs. Meas.* 1 165–178. 7850498

[B73] Watts S. Mackenzie A. Thomas C. Griskaitis A. Mewton L. Williams A. (2013). CBT for depression: a pilot RCT comparing mobile phone vs. computer. *BMC Psychiatry* 13:49. 10.1186/1471-244X-13-49 23391304 PMC3571935

[B74] Webb T. L. Joseph J. Yardley L. Michie S. (2010). Using the internet to promote health behavior change: a meta-analytic review of the impact of theoretical basis, use of behavior change techniques, and mode of delivery on efficacy. *J. Med. Internet Res.* 12 4–23. 10.2196/jmir.1376 PMC2836773 20164043

[B75] Windle G. (2010). What is resilience? A review and concept analysis. *Rev. Clin. Gerontol.* 21 152–169. 10.1017/S0959259810000420

[B76] World Health Organization [WHO] (2011). *mHealth: New Horizons for Health Through Mobile Technologies. Global Observatory for eHealth series - Volume 3.* Geneva: WHO.

[B77] World Health Organization [WHO] (2018). *Mental Health: Strengthening Our Response.* Geneva: WHO.

[B78] Xanthopoulou D. Bakker A. B. Demerouti E. Schaufeli W. B. (2007). The role of personal resources in the job demands-resources model. *Int. J. Stress Manag.* 14 121–141. 10.1037/1072-5245.14.2.121

[B79] Zhao J. Freeman B. Li M. (2016). Can mobile phone apps influence people’s health behavior change? An evidence review. *J. Med. Internet Res.* 18 71–82. 10.2196/jmir.5692 27806926 PMC5295827

