# Supplementary material for: Improving Stress and Positive Mental Health at Work via an App-Based Intervention: A Large-Scale Multi-Center Randomized Control Trial
Source: Front Psychol. 2019 Dec 6;10:2745. doi: 10.3389/fpsyg.2019.02745 (PMC6908507; doi:10.3389/fpsyg.2019.02745)
Supplement: Supplementary file 1 [file Table_1.DOCX]

Supplementary Material

# Controlled model and intensity model

Since the controlled and uncontrolled models showed consistent treatment effects, we focussed on the observed main treatment effects in the uncontrolled model in the main manuscript. Changes introduced by the addition of the control variables, where applicable, are reported in this supplement. Due to selectively missing data, the analyses including the control variables (see Table S1) and number of nights tracked (see Table S2) are based on varying sample sizes.

**General Stress.** We did not observe gender or age effects. However, one of the six trial sites started at elevated baseline (significant site1 main effect), but reported average improvements (site1*time interaction n.s.).

**Cognitive Stress.** The results closely match the development of the general stress scale with significant time and group*time effects. After introduction of the control variables, the time effect became n.s., while two trial sites differed significantly in their baseline, yet did not show a significantly different development (both relative to the grand mean). Again, the main group*time interaction remained significant, i.e., subjects in the ‘Kelaa’ app group improved more relative to the waitlist control group.

**Wellbeing.** Both effects (time and group*time) remained when controlling for age, gender, and trial site. Consistent with the two stress scales, participants at site 1 reported less wellbeing at baseline. Participants at trial site 3 reported significantly less, while participants at trial site 5 reported significantly more change over time compared to the mean rate of change.

**Resilience.** The controlled model yielded a below-average baseline for site 1 (significant site1 main effect).

**Sleeping troubles**. In the controlled model, sleeping troubles remained the only outcome where we observed a significant age effect, with older participants reporting more sleeping troubles. Again, site 1 showed heightened levels of sleeping troubles at baseline compared to the grand mean.

**Social community at work.** We observed a significant gender effect, as females reported higher levels of cooperation than males. Participants’ sense of social community at work trend-significantly increased when controlling for age, gender, and trial site.

**Physical health impairment.** The controlled model yielded an above-average baseline for site 2 (significant site2 main effect), as well as a significant decrease over time (significant site2*time interaction).

*Table S1*

Controlled Hierarchical Linear Models for Fixed Factor Group and Control Variables Age, Gender, and Trial Site (Level 2) over Time (Level 1).

|  | General Stress | Cognitive Stress | Wellbeing | Resilience | Sleeping Troubles | Social Community | Physical Health Impairment |
| --- | --- | --- | --- | --- | --- | --- | --- |
| Fixed Effects | | | | | | | |
| Intercept B (SE) | **2.89 (0.07) ***** | **2.47 (0.08) ***** | **3.24 (0.06) ***** | **5.08 (0.09) ***** | **2.83 (0.10) ***** | **3.84 (0.07) ***** | **2.02 (0.10) ***** |
| Time slope  B (SE) | **-0.11 (0.04) **** | -0.06 (0.03) | **0.10 (0.03) ***** | 0.04 (0.03) | **-0.14 (0.04) **** | -0.02 (0.03) | -0.05 (0.05) |
| Group intercept  B (SE) | -0.02 (0.07) | -0.01 (0.07) | 0.01 (0.06) | -0.05 (0.08) | -0.13 (0.09) | -0.06 (0.07) | -0.09 (0.09) |
| Age intercept  B (SE) | -0.00 (0.00) | -0.00 (0.00) | -0.00 (0.00) | -0.00 (0.00) | **0.01 (0.00) **** | 0.00 (0.00) | 0.01 (0.00) |
| Gender intercept  B (SE) | 0.01 (0.04) | -0.02 (0.04) | 0.04 (0.03) | 0.04 (0.05) | 0.02 (0.06) | **0.11 (0.04) *** | 0.02 (0.06) |
| Site1 intercept  B (SE) | **0.26 (0.12) *** | **0.49 (0.12) ***** | **-0.26 (0.10) **** | **-0.39 (0.14) **** | **0.46 (0.16) **** | -0.18 (0.12) | 0.08 (0.16) |
| Site2 intercept  B (SE) | -0.11 (0.10) | 0.18 (0.11) | 0.15 (0.08) | -0.14 (0.12) | -0.20 (0.13) | 0.17 (0.10) | **0.39 (0.14) **** |
| Site3 intercept  B (SE) | 0.14 (0.08) | 0.11 (0.09) | -0.04 (0.07) | -0.13 (0.10) | -0.02 (0.11) | -0.06 (0.08) | -0.02 (0.11) |
| Site4 intercept  B (SE) | -0.04 (0.10) | -0.02 (0.11) | 0.11 (0.09) | 0.24 (0.13) | -0.10 (0.14) | 0.05 (0.11) | -0.17 (0.15) |
| Site5 intercept  B (SE) | -0.39 (0.27) | **-0.91 (0.29) **** | 0.07 (0.22) | 0.50 (0.33) | -0.14 (0.37) | 0.13 (0.28) | -0.16 (0.38) |
| Group*Time slope  B (SE) | **-0.14 (0.04) ***** | **-0.10 (0.03) **** | **0.07 (0.03) **** | 0.05 (0.03) | -0.06 (0.04) | **0.06 (0.03) *** | 0.05 (0.05) |
| Age*Time slope  B (SE) | 0.00 (0.00) | -0.00 (0.00) | -0.00 (0.00) | 0.00 (0.00) | -0.00 (0.00) | -0.00 (0.00) | 0.00 (0.00) |
| Gender*Time slope  B (SE) | -0.03 (0.02) | -0.01 (0.02) | 0.02 (0.02) | 0.03 (0.02) | -0.01 (0.03) | 0.01 (0.02) | -0.01 (0.03) |
| Site1*Time slope  B (SE) | 0.09 (0.06) | -0.02 (0.06) | -0.02 (0.05) | -0.01 (0.06) | 0.03 (0.07) | -0.03 (0.05) | 0.01 (0.09) |
| Site2*Time slope  B (SE) | -0.02 (0.06) | -0.09 (0.05) | -0.04 (0.04) | 0.01 (0.05) | 0.03 (0.06) | 0.02 (0.05) | **-0.21 (0.08) **** |
| Site3*Time slope  B (SE) | 0.05 (0.04) | 0.01 (0.04) | **-0.08 (0.03) *** | -0.03 (0.04) | 0.04 (0.05) | 0.01 (0.04) | 0.08 (0.06) |
| Site4*Time slope  B (SE) | -0.03 (0.06) | -0.01 (0.05) | -0.04 (0.04) | 0.06 (0.05) | 0.00 (0.07) | -0.02 (0.05) | -0.04 (0.08) |
| Site5*Time slope  B (SE) | -0.08 (0.15) | 0.13 (0.14) | **0.23 (0.11) *** | -0.01 (0.13) | -0.13 (0.18) | -0.02 (0.13) | 0.09 (0.21) |
| Random Effects (Variance Components) | | | | | | | |
| Intercept (SD) | 0.37 (0.61) | 0.47 (0.69) | 0.25 (0.50) | 0.61 (0.78) | 0.73 (0.85) | 0.44 (0.66) | 0.73 (0.85) |
| Time slope (SD) | 0.03 (0.18) | 0.02 (0.15) | 0.01 (0.07) | 0.01 (0.08) | 0.03 (0.17) | 0.02 (0.15) | 0.07 (0.26) |
| Level 1 error (SD) | 0.17 (0.41) | 0.14 (0.38) | 0.11 (0.34) | 0.16 (0.41) | 0.24 (0.49) | 0.12 (0.35) | 0.32 (0.56) |
| Deviance  (k) | 2311.11 (22) | 2229.43 (22) | 1752.51 (22) | 2408.35 (22) | 2797.86 (22) | 2081.32 (22) | 3028.95  (22) |

*Notes*. *N*_Level1_ = 1215-1227, *N*_Level2_ = 484; *k* = number of parameters in model. Time was coded continuously (T1 = 0, T2 = 1, T3 = 2). Group (dummy coded): Waitlist control = 0, ‘Kelaa’ app = 1. Age: Centered around 0, Gender (effect coded): Male = -1, Female = 1, Site (effect coded): Site6 = -1, Site1-Site5 = {0,1}. Method of estimation: full maximum likelihood. The reported estimations are fixed effects with standard errors. Varying degrees of freedom due to selectively missing values. *** *p* < .001, ** *p* < .01, * *p* < .05

*Table S2*

Intensity Model: Longitudinal Hierarchical Linear Models for Fixed Factor Number of Nights Tracked (Level 2: User Metrics) over Time (Level 1).

|  | General Stress | Cognitive Stress | Wellbeing | Resilience | Sleeping Troubles | Social Community | Physical Health Impairment |
| --- | --- | --- | --- | --- | --- | --- | --- |
| Fixed Effects | | | | | | | |
| Intercept B (SE) | **2.99**  **(0.03)***** | **2.61**  **(0.04)***** | **3.25**  **(0.03)***** | **4.97**  **(0.04)***** | **2.75**  **(0.05)***** | **3.85**  **(0.04)***** | **1.99**  **(0.05)***** |
| Time slope  B (SE) | **-0.13**  **(0.02)***** | **-0.11**  **(0.02)***** | **0.06**  **(0.01)***** | **0.06**  **(0.02)**** | **-0.11**  **(0.02)***** | 0.00  (0.02) | -0.01  (0.03) |
| Nights intercept  B (SE) | -0.00  (0.01) | -0.00  (0.01) | -0.00  (0.01) | -0.00  (0.01) | 0.02  (0.01) | -0.01  (0.01) | 0.01  (0.01) |
| Nights*Time slope  B (SE) | **-0.01**  **(0.00)**** | **-0.01**  **(0.00)*** | **0.01**  **(0.00)**** | **0.01**  **(0.00)*** | **-0.02**  **(0.00)***** | **0.01**  **(0.00)*** | -0.01  (0.01) |
| Random Effects (Variance Components) | | | | | | | |
| Intercept (SD) | 0.39 (0.63) | 0.51 (0.72) | 0.27 (0.52) | 0.68 (0.83) | 0.77 (0.88) | 0.45 (0.67) | 0.74 (0.86) |
| Time slope (SD) | 0.03 (0.18) | 0.03 (0.17) | 0.01 (0.08) | 0.02 (0.16) | 0.02 (0.15) | 0.02 (0.16) | 0.07 (0.26) |
| Level 1 error (SD) | 0.17 (0.41) | 0.14 (0.38) | 0.11 (0.33) | 0.17 (0.41) | 0.24 (0.49) | 0.12 (0.35) | 0.33 (0.57) |
| Deviance (k) | 2458.21 (8) | 2399.97 (8) | 1856.71 (8) | 2636.25 (8) | 2931.37 (8) | 2197.29 (8) | 3181.00 (8) |

*Notes*. *N*_Level1_= 1261-1283, *N*_Level2_ = 513-521; *k* = number of parameters in model. Time was coded continuously (T1 = 0, T2 = 1, T3 = 2). Method of estimation: full maximum likelihood. The reported estimations are fixed effects with standard errors. Varying degrees of freedom due to selectively missing values. *** *p* < .001, ** *p* < .01, * *p* < .05
